# Molecular components underlying nongenomic thyroid hormone signaling in embryonic zebrafish neurons

**DOI:** 10.1186/1749-8104-4-20

**Published:** 2009-06-08

**Authors:** Marc A Yonkers, Angeles B Ribera

**Affiliations:** 1 Department of Physiology and Biophysics, University of Colorado Denver at Anschutz Medical Center, Aurora, Colorado 80045, USA; 2 Medical Scientist Training Program, University of Colorado Denver at Anschutz Medical Center, Aurora, Colorado 80045, USA; 3 Neuroscience Program, University of Colorado Denver at Anschutz Medical Center, Aurora, Colorado 80045, USA

## Abstract

**Background:**

Neurodevelopment requires thyroid hormone, yet the mechanisms and targets of thyroid hormone action during embryonic stages remain ill-defined. We previously showed that the thyroid hormone thyroxine (T4) rapidly increases voltage-gated sodium current in zebrafish Rohon-Beard cells (RBs), a primary sensory neuron subtype present during embryonic development. Here, we determined essential components of the rapid T4 signaling pathway by identifying the involved intracellular messengers, the targeted sodium channel isotype, and the spatial and temporal expression pattern of the nongenomic αVβ3 integrin T4 receptor.

**Results:**

We first tested which signaling pathways mediate T4's rapid modulation of sodium current (I_Na_) by perturbing specific pathways associated with nongenomic thyroid hormone signaling. We found that pharmacological blockade of protein phosphatase 1 and the mitogen-activated protein kinase p38 isoform decreased and increased tonic sodium current amplitudes, respectively, and blockade of either occluded rapid responses to acute T4 application. We next tested for the ion channel target of rapid T4 signaling via morpholino knock-down of specific sodium channel isotypes. We found that selective knock-down of the sodium channel α-subunit Na_v_1.6a, but not Na_v_1.1la, occluded T4's acute effects. We also determined the spatial and temporal distribution of a nongenomic T4 receptor, integrin αVβ3. At 24 hours post fertilization (hpf), immunofluorescent assays showed no specific integrin αVβ3 immunoreactivity in wild-type zebrafish embryos. However, by 48 hpf, embryos expressed integrin αVβ3 in RBs and primary motoneurons. Consistent with this temporal expression, T4 modulated RB I_Na _at 48 but not 24 hpf. We next tested whether T4 rapidly modulated I_Na _of caudal primary motoneurons, which express the receptor (αVβ3) and target (Na_v_1.6a) of rapid T4 signaling. In response to T4, caudal primary motoneurons rapidly increased sodium current peak amplitude 1.3-fold.

**Conclusion:**

T4's nongenomic regulation of sodium current occurs in different neuronal subtypes, requires the activity of specific phosphorylation pathways, and requires both integrin αVβ3 and Na_v_1.6a. Our *in vivo *analyses identify molecules required for T4's rapid regulation of voltage-gated sodium current.

## Background

Although thyroid hormone deficiency results in severe neurodevelopmental deficits [1], the underlying mechanisms remain unclear. The traditional mechanism for thyroid hormone action involves conversion of secreted thyroxine (T4) to triiodothyronine (T3) by deiodination at the cellular level by target tissues. T3 then binds to intracellular nuclear thyroid hormone receptors to modulate transcription over a time course of hours to days [2,3]. However, deletion of nuclear thyroid hormone receptors have little effect on development [4], suggesting that either unliganded thyroid hormone nuclear receptors mediate the consequences of hypothyroidism [5] or non-nuclear thyroid hormone receptors remain functional.

Recent studies have shown that exogenously applied T3 and T4 can act through extranuclear plasma membrane receptors on a timescale of minutes [6], providing a nongenomic mechanism for thyroid hormone signaling apart from traditional nuclear signaling. Bergh *et al*. [7] showed that the integrin dimer αVβ3 acts *in vivo *as a nongenomic thyroid hormone receptor in the chick chorioallantoic membrane and that T4-αVβ3 binding regulates angiogenesis. In addition, they found that αVβ3 displayed a higher binding affinity for T4 over T3. The increased specificity for T4 supports the view that T4 acts as more than a prohormone to T3.

Integrins are present during nervous system development [8] and regulate neuronal migration [9] and apoptosis [10]. We previously reported that blockade of integrin αVβ3 reduced voltage-gated sodium current in Rohon-Beard primary sensory neurons (RBs) [11]. Here, we focus on the intracellular pathways that translate T4-αVβ3 signaling into modulation of sodium current (I_Na_). Davis and colleagues [7,12] demonstrated that T4 binding to integrin αVβ3 activates the mitogen-activated protein kinase (MAPK) extracellular regulated kinase (ERK1/2) pathway. In addition, thyroid hormones can regulate other second messenger pathways, including the MAPK p38 isoform [13] and protein kinase C [14,15]. The candidate intracellular messengers of rapid thyroid hormone signaling may regulate sodium channel function via phosphorylation.

One possible scenario is that the involved intracellular kinases and phosphatases directly regulate the phosphorylation state of a sodium channel. Consistent with this possibility, phosphorylation of voltage gated sodium channels by MAPK (p38) reduces I_Na _amplitude by 50% [16]. In the zebrafish embryo, MAPK (ERK1/2), MAPK (p38), and protein phosphatase (PP) subtypes PP1 and PP2A are all expressed in the spinal cord at 48 hours post-fertilization (hpf) [17], allowing for pharmacological assay of the effects of kinase and phosphatase inhibition on RB I_Na _and embryonic T4 signaling.

Regardless of whether phosphorylation directly targets sodium channels, our data indicate that rapid T4 signaling regulates sodium channel function. In RBs, two different types of sodium channels, Na_v_1.1l and Na_v_1.6a, carry I_Na _[18]. The contribution of the two channel types to RB I_Na _changes during development, with Na_v_1.6a channels accounting for a majority of RB current at 48 hpf. We previously found I_Na _sensitivity to T4 at 48 hpf [11], raising the possibility that T4 rapidly regulates Na_v_1.6a channels. While Na_v_1.6a is the major contributor to RB I_Na_, it is also widely expressed in the nervous system and is of critical importance to development [19]. T4 regulation of Na_v_1.6a current would provide a mechanism for thyroid hormone to serve as an important developmental regulator of neural activity.

Here, we identify the signaling mechanisms and sodium channels underlying nongenomic T4 activity in embryonic zebrafish neurons. We also define the temporal and spatial expression pattern of the nongenomic T4 receptor, integrin αVβ3, in zebrafish embryos. Our results indicate that neuronal cell types expressing both αVβ3 and Na_v_1.6a sodium channels respond rapidly to T4 with an increase in I_Na _amplitude.

## Materials and methods

All experimental procedures were approved by the Animal Care and Use Committee of the Center for Comparative Medicine at the University of Colorado Denver – Anschutz Medical Campus.

### Animals

Zebrafish (*Danio rerio*) adults were bred according to guidelines outlined in The Zebrafish Book [20]. Embryos were incubated at 28.5°C in embryo medium (130 mM NaCl, 0.5 mM KCl, 0.02 mM Na_2_HPO_4_, 0.04 mM KH_2_PO_4_, 1.3 mM CaCl_2_, 1.0 mM MgSO_4_, 0.4 mM NaH_2_CO_3_) and staged according to external morphology [21].

### Electrophysiology

Whole cell voltage clamp recordings were obtained from zebrafish spinal cord RBs as previously described [11,18,22]. Voltage clamp recordings from caudal primary motoneurons (CaPs) were obtained from the Tg(hb9:GFP) line (gift of Drs Michael Fox and Joshua Sanes, Harvard University, Cambridge, MA, USA) that express green fluorescent protein (GFP) in motoneurons [23]. Tg(hb9:GFP) zebrafish were immobilized in Ringer solution (145 mM NaCl, 3 mM KCl, 1.8 mM CaCl_2_, and 10 mM HEPES, pH 7.2) containing 0.02% tricaine (Sigma St Louis, Missouri, USA) and glued laterally to glass coverslips. Glass dissecting needles sufficed for removal of skin and detachment of overlying muscle fibers. Muscle fibers and secondary motoneurons were removed by a suction pipette to expose primary motoneurons. Three properties identified CaPs: GFP expression, cell body size (approximately 10 μM diameter), and ventrally projecting axons [24]. For initial experiments, we used a reduced extracellular sodium bath solution (30 mM NaCl, 97 mM N-methyl glucamine, 20 mM tetraethylammonium (TEA), 3 mM KCl, 2 mM CoCl_2_, and 10 mM HEPES) to reduce potential series resistance voltage errors arising from large I_Na _amplitudes. However, some experimental manipulations (for example, knockdown of sodium channel α-subunits or phosphatase blockade) reduced I_Na _amplitudes; in these cases, we used a normal 125 mM extracellular sodium concentration to increase I_Na _amplitudes and the sensitivity of our measurements. Glass electrodes (2.0 to 3.5 MÙ) were filled with solution containing 10 mM NaCl, 135 mM CsCl, 10 mM EGTA, and 10 mM HEPES. We subtracted passive leak currents and capacitive transients from recordings of voltage-gated sodium using a P/8 protocol. Data were acquired using an Axopatch 200B amplifier (Axon Instruments, Foster City, California, USA) and analyzed with Clampfit8 (Axon Instruments) and Origin software (OriginLab, Northampton, Massachusetts, USA).

### Data presentation

Results are presented as means ± standard errors. Statistical analysis was performed with Origin v7.0 software (OriginLab). Statistical comparisons of means were performed by one-way ANOVAs with Bonferroni corrections for multiple comparisons.

### Hormone and drug application

T4 (3,3',5,5'-tetraiodo-L-thyronine (thyroxine); Sigma) was prepared as a 30 mM stock solution in dimethyl sulfoxide (DMSO) that was diluted to final concentrations in extracellular recording solution immediately before use. Vehicle (DMSO) control experiments indicated that the final concentration of DMSO (0.001%) had no effect on I_Na _amplitudes; therefore, control and vehicle control data were pooled (Control/DMSO). Kinase and phosphatase inhibitors were applied to neurons in semi-intact preparations of the zebrafish embryo after obtaining control recordings prior to treatment. PD98059 (50 μM; Sigma), 1 μM SB203580 (Sigma), or okadaic acid (OA; 1 nM to 1 μM; Sigma) was applied for 1 hour at room temperature before obtaining post-treatment recordings. The drugs remained in the bath during post-treatment recording.

### Immunocytochemistry

Whole mount embryos (24 to 48 hpf) were processed for immunocytochemistry as previously described [25]. The primary antibody, mouse anti-human monoclonal LM609 (Millipore, Billerica, MA, USA), was diluted 1:100. Secondary antibody was applied overnight at 4°C (1:500; goat anti-mouse conjugated to Alexa 568; Invitrogen-Molecular Probes, Carlsbad, California, USA). Controls consisted of experiments done with LM609 that had been previously incubated with 50 μg/ml human αVβ3 (Millipore). In some experiments, the Tg(isl3:GFP) line (gift of Drs Andrew Pittman and Chi-Bin Chien, University of Utah) expressing GFP in RBs or the Tg(hb9:GFP) line expressing GFP in motoneurons were used. GFP expression was revealed using a rabbit anti-GFP antibody conjugated to Alexa 488 (1:400; Invitrogen). For analysis, embryos were mounted in a 1% low melting point agarose solution and imaged using a Zeiss Pascal Confocal Microscope using 10× or 40× objectives and separate 488 and 568 laser lines. Fluorescent images were collected digitally as z-stacks of 2 μm slices. Data are presented as projections of 20 to 25 slices.

### Morpholino knock-down

Antisense oligonucleotide morpholinos (MOs) targeting sodium channels Na_v_1.6a (1.6 MO) and Na_v_1.1l (1.1 MO) were synthesized and prepared as previously described [18]. Injection solutions contained the dye Fast Green (1%) to report efficient delivery of the MO to animal cells. For each Na_v_1 MO, control MOs were synthesized by introducing mismatches at five positions. Embryos that had either Na_v_1.6a or Na_v_1.1l sodium channel subunit knock-down were created by injection of 2 to 3 hpf wild-type embryos with solution containing 0.3 mM MO antisense oligonucleotide [18]. All MOs have been used previously and tested by standard control experiments [18,19,26].

Embryos that had Fast Green within the animal cell 15 minutes post-injection were transferred to a petri dish containing embryo medium (130 mM NaCl, 0.5 mM KCl, 0.02 mM Na_2_HPO_4_, 0.04 mM KH_2_PO_4_, 1.3 mM CaCl_2_, 1.0 mM MgSO_4_, 0.4 mM NaH_2_CO_3_) and then raised at 28°C until 48 hpf. Embryos that were injected with the 1.6 MO or 1.1 MO and selected for recording are referred to as Na_v_1.6a or Na_v_1.1l morphants, respectively.

## Results

### Blockade of either p38 MAPK or PP1 alters RB I_Na _amplitude and occludes the rapid T4 effect

In other systems, rapid thyroid hormone signaling involves intracellular signaling kinase pathways such as MAPK (ERK1/2) and MAPK (p38) [7,12,13,27-29]. To identify intracellular mediators of rapid T4 signaling in RBs, we used a pharmacological approach. We inhibited MAPK (ERK1/2) or MAPK (p38) signaling by using the blockers PD98059 or SB203580, respectively. In addition, because phosphatase effects oppose kinase action we used OA to block serine/threonine phosphatases. At low OA concentrations we blocked PP2A (1 to 20 nM OA) and at higher concentrations we blocked both PP2A and PP1 (1 μM OA). After incubation of spinal cord preparations in conditions of kinase or phosphatase blockade, we tested for effects of kinase and phosphatase inhibitors on I_Na _amplitude, in the absence and presence of T4.

**Figure 1:**
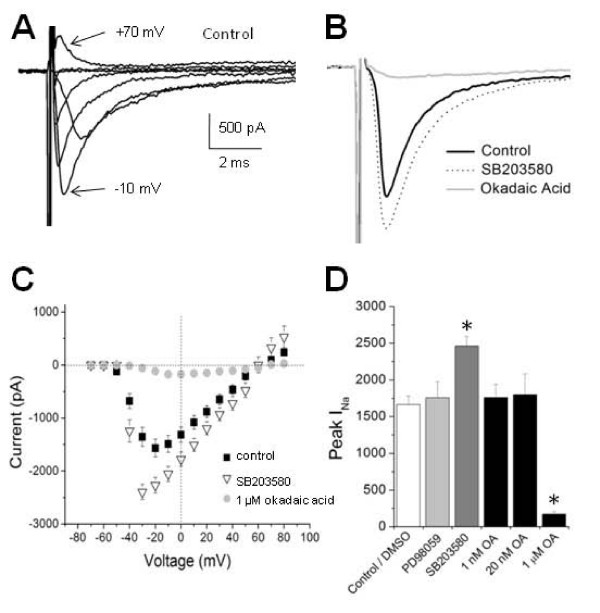
**p38 and protein phosphatase 1 blockade altered tonic sodium current peak amplitudes**. **(A) **I_Na _was elicited by depolarizing voltage steps ranging between -70 and +70 mV in 125 mM extracellular sodium solution from a holding potential of -80 mV. Traces represent recordings obtained from a Rohon-Beard cell (RB) sensory neuron in a 50 hpf embryo. **(B-D) **To block relevant kinase pathways, we incubated the exposed zebrafish spinal cord preparation in the indicated inhibitors for 1 hour prior to recording I_Na _peak amplitudes. We blocked two separate kinase pathways, ERK1/2 and p38, with the inhibitors PD98059 and SB203580, respectively. In order to block protein phosphatase (PP) activity, we used two different okadaic acid (OA) concentrations that inhibit either PP2A only (1 to 20 nM), or both PP2A and PP1 (1 μM). (B) After a 1 hour incubation in SB203580, RB I_Na _peak amplitude increased. Conversely, 1 μM OA decreased RB I_Na _peak amplitude. (C) Average current-voltage (I-V) relationships for I_Na _recorded after p38 and PP1 inhibition showed changes in I_Na _amplitude without alteration of the reversal potential (E_rev_). p38 inhibition (n = 14) led to an increase in I_Na _amplitude compared to controls (n = 23). In contrast, phosphatase inhibition with 1 μM OA (n = 7) reduced I_Na _compared to controls. Neither treatment affected E_rev_. (D) Peak I_Na _amplitudes of cells exposed to the ERK1/2 inhibitor PD98059 (n = 16) did not significantly change I_Na _peak amplitude compared to controls (n = 23). However, incubation of zebrafish embryos in the p38 inhibitor SB203580 (n = 14) significantly increased I_Na _peak amplitude (*P *< 0.05; ANOVA). Inhibition of PP2A (1 and 20 nM OA; n = 6 and 11, respectively) did not significantly change peak I_Na _amplitude compared to controls. However, 1 μM OA (n = 7), which inhibits both PP2A and PP1, resulted in a significant reduction in I_Na _peak amplitude (*P *< 0.05; ANOVA). Asterisks represent a p-value of < 0.05 and error bars represent standard errors.

**Figure 2:**
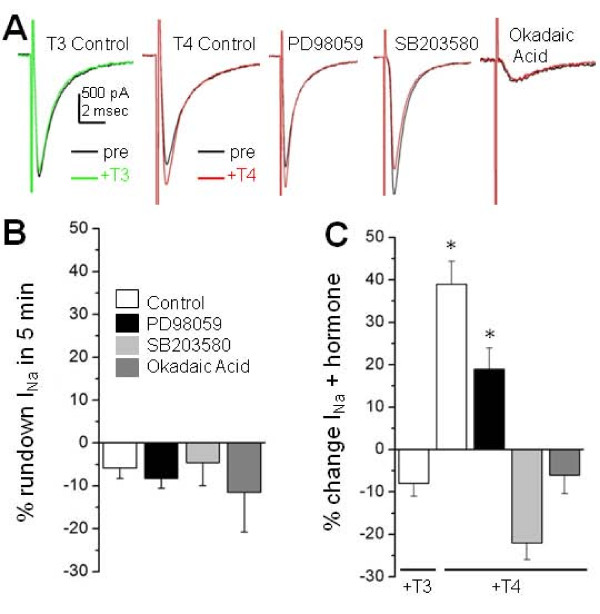
**Blockade of p38 or protein phosphatase 1 occluded T4-induced rapid increase in sodium current amplitude**. **(A) **The traces show typical Rohon-Beard cell (RB) I_Na _recordings elicited by a -10 mV depolarizing voltage step before (black) and after acute application of 30 nM T3 (green) or 30 nM T4 (red). The PD98059, SB203580, and okadaic acid (OA) treatment groups present RB I_Na _recorded after 1 hour drug incubation. **(B) **Changes in I_Na _peak amplitude over 5 minutes for either control, PD98059, SB203580, or OA showed that none of the treatments increased run-down of the current during the recording period. **(C) **Acute application of 30 nM T3 (n = 5) did not alter RB I_Na _compared to control cells unexposed to acute hormone treatment. However, 30 nM T4 (n = 11) significantly increased peak I_Na _amplitude over time in control RBs. PD98059 treatment (n = 6) did not significantly affect T4's increase in peak I_Na _amplitude compared to controls. However, SB203580 (n = 5) or OA (n = 3) treatments prevented a rapid increase in I_Na _amplitude (*P *< 0.05; ANOVA) in response to acute T4 application. The data presented were acquired in the presence of 125 mM extracellular Na^+^. Asterisks represent a p-value of < 0.05 and error bars represent standard errors.

Control/DMSO cells displayed a I_Na _peak amplitude of 1,665 ± 116 pA (n = 23; Figure 1D) and responded to acute T4 application with a 39 ± 5% increase in amplitude (n = 4; *P *< 0.05). To test for involvement of ERK1/2, we used PD98059 (50 μM), a specific inhibitor of the ERK 1/2 pathway component MEK1 [30,31]. By itself, PD98059 did not significantly alter I_Na _peak density (1,756 ± 217 pA; n = 16; *P *= 0.69) compared to control/DMSO (Figure 1D). Further, RBs exposed to PD98059 could still respond to T4 by rapidly increasing I_Na _amplitude (Figure 2A, C). Compared to control/DMSO cells, however, PD98059-treated cells showed a blunted response to T4 (Figure 2C).

Whereas the PD98059 results suggest that the ERK1/2 pathway may partially mediate the rapid effects of T4, the data do not implicate ERK1/2 signaling in tonic regulation of the number of available sodium channels. In contrast, nongenomic T4 signaling regulates both the rapid response to T4 as well as the tonic levels of available sodium channels in RBs [11]. Overall, the PD98059 results suggest minimal involvement of the ERK1/2 pathway in T4 regulation of RB I_Na_.

We next tested the contribution of the MAPK (p38) pathway that mediates rapid thyroid hormone signaling triggered by T3 [13] and regulates I_Na _density [16]. In contrast to blockade of the ERK1/2 pathway, pharmacological inhibition of the p38 pathway increased tonic I_Na _amplitudes (in the absence of exogenous T4) 1.48-fold (2458 ± 134 pA; n = 14; *P *< 0.05, ANOVA) (Figure 1D). Moreover, following SB203580 treatment, T4 no longer produced a rapid increase in I_Na _amplitude. In fact, after SB203580 treatment, T4 application led to a 22 ± 4% (n = 5) decrease in RB I_Na _amplitude (Figure 2A, C). Overall, the effects of SB203580 support involvement of the p38 pathway in regulation of both the resting levels of available RB sodium channels as well as the rapid response of RB I_Na _to T4.

The SB203580 results suggest that T4 acts rapidly on RBs by opposing ongoing MAPK p38 signaling either by inhibiting the kinase or by activating relevant serine/threonine phosphatases. Two serine/threonine phosphatases, PP2A and PP1, are both ubiquitously expressed in the zebrafish spinal cord at 48 hpf [17], a time when T4 rapidly regulates RB sodium current. To test for involvement of PP2A or PP1, we incubated zebrafish spinal cords with the serine/threonine phosphatase inhibitor OA prior to recording I_Na _from RBs. At concentrations of 1 to 20 nM, OA specifically inhibits PP2A. However, 1 and 20 nM OA did not significantly alter I_Na _peak amplitudes in the absence of T4 (1,761 ± 179 pA (n = 6) and 1,798 ± 283 pA (n = 11), respectively). In contrast, the IC_50 _for PP1 inhibition by OA is much higher (approximately 0.5 μM) [32]. OA at 1 μM produced a drastic 90% reduction in resting RB I_Na _peak amplitudes (169 ± 33 pA; n = 7; *P *< 0.00001, ANOVA; Figure 1D). Further, following 1 μM OA treatment, T4 no longer increased RB I_Na _amplitude (-6 ± 4% change; n = 7; *P *= 0.98 versus no T4 added; Figure 2A, C). Both the tonic reduction in I_Na _peak amplitude and the occlusion of rapid T4 effects under conditions of PP1 blockade support the view that PP1 activity modulates RB I_Na _amplitudes.

### Rapid T4 effects require sodium channel α-subunit Na_v_1.6a

We tested whether T4's rapid action on RB I_Na _amplitude targeted a specific voltage-gated sodium channel isotype. RBs express two different sodium channel α-subunit genes, *scn1.1l *and *scn8aa*, which code for the voltage-gated sodium channel proteins Na_v_1.1l and Na_v_1.6a, respectively [33]. Interestingly, the conductance carried by the mammalian homologue of Na_v_1.6a is significantly reduced by activation of MAPK p38 [16]. We knocked down either Na_v_1.1l or Na_v_1.6a using MOs, as done previously for effective and selective elimination of specific Na_v_1 proteins in the zebrafish embryo [18,19].

**Figure 3:**
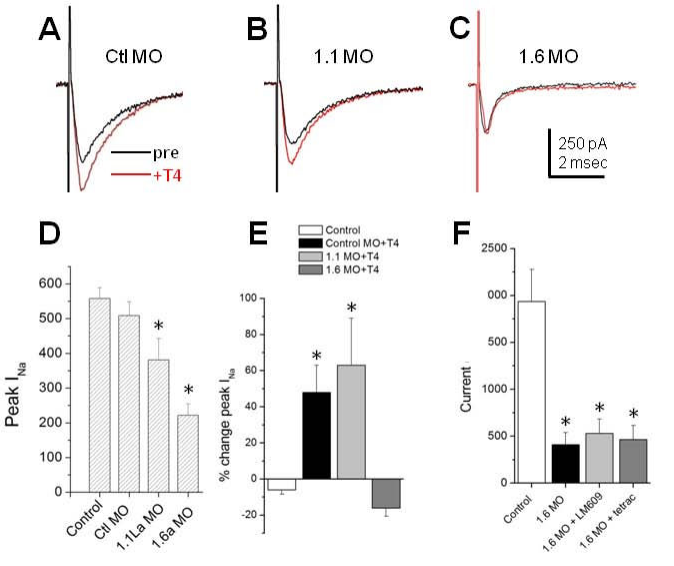
**Rohon-Beard cells in Na_v_1.6a morphants did not show a rapid T4 response**. **(A-C) **The representative traces show Rohon-Beard cell (RB) I_Na _before (black) and 5 minutes after (red) acute T4 application. Embryos had been injected with either the 1.6 missense control morpholino (Ctl MO) (A), or MOs targeting sodium channels Na_v_1.1l (1.1 MO) or Na_v_1.6a (1.6 MO) (C). **(D) **Injection of 1.6 MO or 1.1 MO reduced RB I_Na _peak amplitude compared to control. The reductions in I_Na _peak amplitudes were consistent with previously reported values for successful knockdown of either Na_v_1.6a or nav1.1la [18]. Injection of 5-missense control MO did not significantly alter I_Na _peak amplitude. The data presented in (D) were recorded in the presence of 30 mM extracellular Na^+^. **(E) **Embryos injected with 5-missense MO or 1.1 MO showed significant increases in RB I_Na _peak amplitude after 5 minutes of T4 application. In contrast, RBs in 1.6 morphant embryos did not show an increase in I_Na _amplitude after T4 application. **(F) **Injection of 1.6 MO (n = 3) reduced peak I_Na _amplitude in 125 mM extracellular recording solution compared to controls (n = 5; *P *< 0.05). In control embryos, thyroid hormone antagonism (tetrac) or αVβ3 blockade (LM609) injection reduced I_Na _amplitudes [11]. However, in 1.6 morphant embryos, neither LM609 (n = 5) nor tetrac (n = 9) altered I_Na _amplitudes compared to 1.6 morphants unexposed to LM609 or tetrac (n = 3). Asterisks represent a p-value of < 0.05 and error bars represent standard errors.

As found previously, knockdown of either Na_v_1.6a or Na_v_1.1l α-subunits led to decreased RB I_Na _peak amplitudes (Figure 3D) [18]. Further, injection of the control Na_v_1.6a 5-missense MO did not significantly alter RB I_Na _peak density. We next tested whether knock-down of either sodium channel subunit prevented T4's rapid modulation of RB I_Na _by acute application of T4 to morphant embryos. We found that T4 increased RB I_Na _amplitude in control morphants (45 ± 8%; *P *< 0.05 versus no hormone) to a similar extent as in wild-type embryos (39 ± 5%) [11] (Figure 3A, E). However, in Na_v_1.6a morphants, T4 application did not increase I_Na _amplitudes (-16 ± 5%; *P *< 0.05 versus control morphants), indicating that rapid T4 action targets Na_v_1.6a channels.

In contrast, in Na_v_1.1l morphant embryos, T4 application increased RB I_Na _(63 ± 10%; *P *< 0.05 versus no hormone), suggesting that T4 does not require Na_v_1.1l channels to increase RB I_Na _density. In Na_v_1.1l morphants, the increase in I_Na _amplitude induced by T4 actually exceeded that produced in controls (63 ± 10% versus 45 ± 8%; *P *< 0.05), presumably because Na_v_1.6a channels carry the majority if not all of the remaining current [18]. The effects of T4 on Na_v_1.6a and Na_v_1.1la morphants indicate that rapid T4 signaling targets Na_v_1.6a sodium channels.

### Antagonists of nongenomic thyroid hormone signaling do not affect RB I_Na _in Na_v_1.6a morphants

Antagonists of thyroid hormone (3,3',5,5'-tetraiodothyroacetic acid (tetrac)) or of the integrin αVβ3 block rapid T4 signaling in RBs [11]. We next tested whether the effects of thyroid hormone antagonism or αVβ3 blockade targeted Na_v_1.6a-mediated current. We exposed Na_v_1.6a morphants to the thyroid hormone analog tetrac, or the αVβ3 function blocking antibody LM609. To more readily detect changes in RB I_Na _amplitude in Na_v_1.6a morphants, we raised the extracellular sodium concentration to 125 mM to increase I_Na _amplitudes. We previously showed that tetrac reduces RB I_Na _amplitudes in wild-type embryos [11]. However, in Na_v_1.6a morphants, tetrac did not significantly change RB I_Na _amplitudes compared to Na_v_1.6a morphants unexposed to tetrac (Figure 3F). We had also previously demonstrated that LM609 reduced RB I_Na _amplitudes by 46% in wild-type embryos [11]. In contrast, in Na_v_1.6a morphants, LM609 injection did not significantly affect I_Na _peak amplitudes compared to uninjected Na_v_1.6a morphants (Figure 3F). The lack of effect of either tetrac or LM609 in Na_v_1.6a morphants further supports that the rapid T4-integrin signaling pathway specifically targets Na_v_1.6a channels.

### Developmental regulation of αVβ3 expression temporally restricts T4 signaling in RBs

Whether T4 induced increases in sodium current occur throughout development or if T4 signaling begins at a defined developmental stage is unknown. The above results combined with our previous study [11] indicate that in order to respond rapidly to T4 at 48 hpf, RBs require integrin αVβ3 and the Na_v_1.6a sodium channel α-subunit. We next determined whether αVβ3 is present and if RBs respond rapidly to T4 at earlier stages. We reasoned that absence of integrin αVβ3 would prevent T4 from rapidly modulating RB I_Na_. Accordingly, we tested our prediction by determining the spatial and temporal αVβ3 expression pattern.

**Figure 4:**
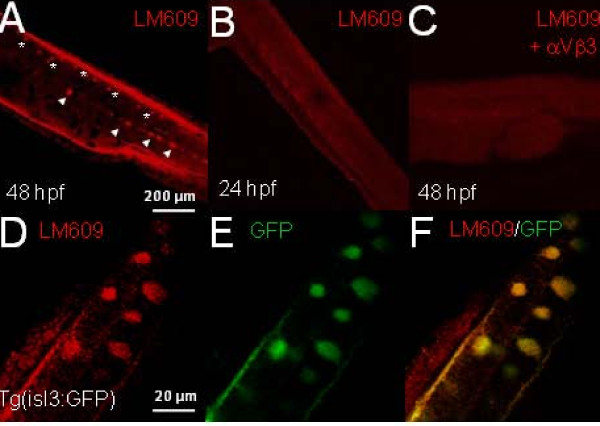
**Zebrafish embryos expressed integrin αVβ3 at 48 hpf but not at 24 hpf**. **(A) **The 48-hpf zebrafish embryos were fixed and incubated with the LM609 antibody. LM609 immunoreactivity was revealed using a rhodamine-labeled fluorescent secondary antibody and preparations were examined using confocal imaging. At 48 hpf, LM609 immunoreactivity specifically labeled cells along the dorsal spinal cord (asterisks), the location of Rohon-Beard cells (RBs). LM609 also labeled cells in the ventral spinal cord (arrowheads), consistent with the location of motoneurons. **(B) **In contrast, 24-hpf embryos did not display LM609 immunoreactivity, consistent with an absence of integrin αVβ3 expression at 24 hpf. **(C) **Pre-incubation of LM609 with 50 μg/ml αVβ3 protein blocked immunoreactivity in 48 hpf embryos, indicating that the labeling was specific for αVβ3. **(D) **Tg(isl3:GFP) transgenic zebrafish embryos expressed green fluorescent protein (GFP) in RBs under control of the *islet3 *promoter. **(E) **LM609 immunoreactivity was present in dorsal cells. **(F) **GFP and LM609 immunoreactivity colocalized in RB soma and axon tracts. Scale bars: 200 μm (A-C); 20 μm (D, E, F).

To determine when RBs express the plasma membrane T4 receptor, we performed immunocytochemistry using the LM609 antibody that specifically detects the αVβ3 dimer [34]. As expected, at 48 hpf the LM609 antibody revealed immunoreactivity in dorsal spinal cord cells (Figure 4A) and cells in the ventral spinal cord. However, at 24 hpf, no immunoreactivity was detected (Figure 4B). To test whether immunoreactivity was specific for αVβ3, we pre-incubated LM609 with integrin αVβ3 protein prior to zebrafish application. We found pre-incubation of LM609 with αVβ3 protein prevented detection of immunoreactivity (Figure 4C), indicating specificity of immunostains for αVβ3.

LM609 immunoreactive cells localized to the dorsal spinal cord where RBs reside. To identify LM609 immunoreactive cells as RBs, we used transgenic Tg(isl3:GFP) embryos. In this line, the *isl3 *promoter drives GFP expression in RBs (A Pittmann and Chi-Bin Chien, personal communication). In 48 hpf Tg(isl3:GFP) embryos (Figure 4D–F), LM609 immunoreactivity colocalized with GFP, revealing αVβ3 expression on RB bodies. This result is consistent with rapid αVβ3-dependent T4 signaling in 48 hpf RBs [11]. In contrast, at 24 hpf, zebrafish embryos did not show specific LM609 immunolabeling in either the dorsal or ventral spinal cord (Figure 4B). These data indicate that αVβ3 dimers appear on RBs after 24 hpf.

**Figure 5:**
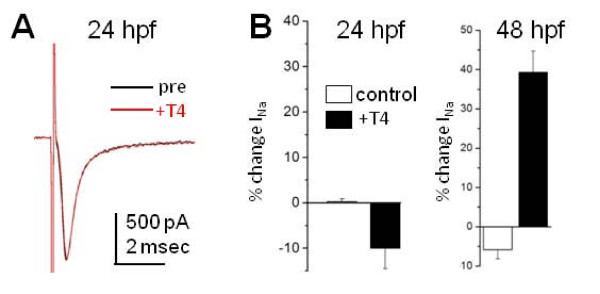
**T4 did not affect Rohon-Beard cell sodium current amplitude at 24 hpf**. **(A) **The 24 hpf zebrafish embryos were tested for the ability of Rohon-Beard cells (RBs) to respond rapidly to T4. I_Na _peak amplitudes did not significantly change over 5 minutes of T4 application. In the representative recording, I_Na _was elicited by a depolarizing voltage step to -10 mV from a holding potential of -80 mV. **(B) **At 24 hpf, 30 nM T4 application did not significantly alter RB I_Na _(n = 6) compared to controls (n = 4). In contrast, when αVβ3 is expressed in the spinal cord at 48 hpf, T4 acutely increased RB I_Na _(48 hpf data from [11]).

At 24 hpf, Na_v_1.6a underlies a portion of RB I_Na _[18]. Nonetheless, according to our model, the lack of αVβ3 expression on RBs at 24 hpf would prevent T4 from rapidly modulating RB I_Na _To test this prediction, we acutely applied 30 nM T4 to the spinal cords of 24 hpf embryos and recorded RB I_Na_. In contrast to results obtained from 48 hpf embryos [11], we found that T4 had no significant effect on RB I_Na _amplitude at 24 hpf (Figure 5A, B). These results indicate that acute modulation of RB I_Na _requires αVβ3.

### T4 rapidly increases sodium current density in CaPs

**Figure 6:**
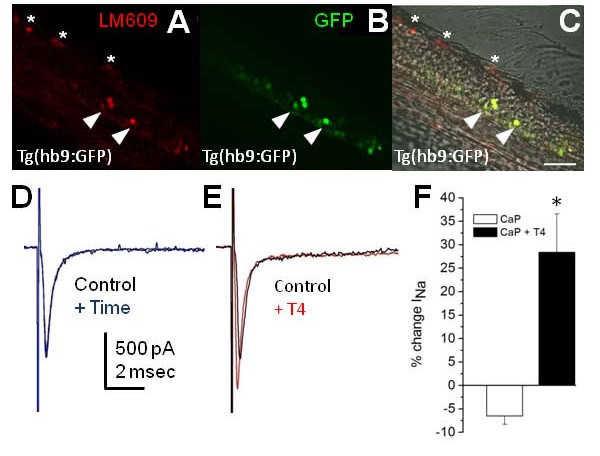
**T4 increases sodium current in caudal primary motoneurons**. **(A-C) **Tg(hb9:GFP) transgenic embryos were incubated with the αVβ3 antibody LM609. The 48-hpf Tg(hb9:GFP) transgenics displayed LM609 immunoreactivity in dorsal (asterisks) and ventral cells (arrowheads). The ventral immunoreactivity for LM609 colocalized with green fluorescent protein (GFP) in primary motoneurons (C, arrowheads). Images are oriented with dorsal neurons and ventral neurons in the upper left and lower right corners, respectively. Scale bar: 50 μm. **(D, E) **Caudal primary motoneuron (CaP) I_Na _was recorded for 5 minutes (+ Time) either in the absence (D) or presence of T4 (E). Each trace shows current in response to a -10 mV depolarizing stimulus. **(F) **At 50 to 55 hpf, zebrafish CaPs showed rapid increases in I_Na _amplitude in response to acute application of T4. The 30 nM T4 application significantly increased CaP I_Na _(n = 5; *P *< 0.01) compared to controls (n = 5).

As another test of the requirement for integrin αVβ3, MAPK p38/PP1, and the Na_v_1.6a sodium channel α-subunit for rapid T4 signaling, we sought to identify another neuronal cell type that expressed these critical components and test whether T4 could also rapidly modulate I_Na _amplitude. The intracellular messengers are ubiquitously expressed in the zebrafish spinal cord [17], and several ventral spinal cord neurons, including interneurons and motoneurons, express Na_v_1.6a [19,33]. We detected LM609 immunoreactivity in the ventral spinal cord (Figure 4A) and now determined the identity of these neurons by using the Tg(hb9:GFP) line. In Tg(hb9:GFP) embryos, motoneurons express GFP, allowing morphological assessment of cell bodies and axonal projections [23]. At 48 hpf, a subset of GFP expressing cells in Tg(hb9:GFP) embryos were also immunoreactive for LM609 (Figure 6A–C). In particular, ventral neurons with large diameter cell bodies, a hallmark of primary motoneurons, were co-positive for GFP and LM609 (Figure 6B, C).

At 48 hpf, the spinal cord contains three different types of primary motoneurons [24]. One primary motoneuron, CaP, expresses Na_v_1.6a at 48 hpf [19,33]. Because rapid T4 modulation of I_Na _targets Na_v_1.6a, co-expression of Na_v_1.6a and integrin αVβ3 raised the possibility that CaPs might respond to T4 with a rapid increase in I_Na _amplitude. To test this possibility, we applied 30 nM T4 to CaP motoneurons, identified in Tg(hb9:GFP) 48-hpf embryos by cell body size and ventrally projecting axons, while recording I_Na_. In control CaP recordings, I_Na _peak density decreased over 5 minutes by 7 ± 2%. However, CaP I_Na _density significantly increased by 28 ± 8% (*P *< 0.005) after acute T4 application (Figure 6D–F). These results support our model that rapid regulation of I_Na _density by T4 requires αVβ3 and targets Na_v_1.6a channels.

## Discussion

### Summary

Our results identify messengers and targets of a rapid thyroid hormone signaling pathway that functions in the zebrafish embryonic nervous system. The T4 pathway rapidly induces increased sodium current amplitudes and requires the sodium channel isotype Na_v_1.6a even though neurons express several different sodium channel isotypes. Moreover, the results suggest that the phosphorylation state of an involved protein, perhaps even the targeted sodium channel isotype, determines sodium channel activity.

### Signaling pathway

We propose a model in which the phosphorylation status of a particular protein or set of proteins regulates RB I_Na _amplitude, and T4 rapidly alters the phosphorylation status of relevant protein(s). Further, the data suggest that serine/threonine phosphorylation and dephosphorylation reduce and increase, respectively, RB I_Na _amplitude. To increase RB I_Na _amplitude, T4 may rapidly activate PP1 and/or inhibit p38. Consistent with our findings, PP1 modulates I_Na _amplitudes in rat striatal neurons [35], which express Na_v_1.6 [36]. Schiffmann *et al*. [35] found that PP1 blockade reduced rat striatal I_Na _amplitudes, similar to our results in zebrafish RBs.

Our data do not provide information about the identity of the phosphorylated protein(s). One possibility is that T4 binding to αVβ3 activates intracellular pathways that directly phosphorylate sodium channel α-subunits. In ND7/23 cells transfected with mammalian Na_v_1.6 sodium channels, activation of p38 produces a decrease in I_Na _[16]. Of particular relevance, biochemical analysis demonstrated that p38 activity regulated phosphorylation of a specific Na_v_1.6 serine, S553, revealing the Na_v_1.6 sodium channel as a direct p38 phosphorylation target [16]. On this basis, the RB T4-αVβ3 pathway may modulate I_Na _by regulating the phosphorylation state of the conserved serine residue in zebrafish Na_v_1.6a.

One result that the model does not fully account for, however, is the large reduction in RB I_Na _amplitude produced by PP1 inhibition. We previously reported that either T4 or αVβ3 blockade reduced I_Na _by only 50%, yet 1 μM OA reduced RB I_Na _by nearly 90%. This discrepancy could be attributed to different degrees of PP1 inhibition by 1 μM OA versus T4/αVβ3 blockade. The large decrease in I_Na _amplitude produced by 1 μM OA could also reflect phosphorylation effects triggered by non-T4-dependent mechanisms. For example, protein kinases C and A also have effects on Na_v_1.6 amplitude [37].

### αVβ3 acts as a T4 receptor in the nervous system

The important developmental roles of integrins as cell surface adhesion proteins have been well studied [8]. Less-well studied, however, is the potential role of integrins as receptors for hormones. Because neurons and glia [38] express αVβ3, nongenomic T4 signaling via αVβ3 may play an important role in nervous system development.

Here, we focused on integrin's role as a plasma membrane receptor for thyroid hormones that traditionally signal through nuclear receptors. We focused on the integrin dimer, αVβ3, a protein that is important for neuronal migration and axon extension [39-41], and is expressed on dorsal root ganglia [42,43]. The fact that RGD (Asp-Gly-Arg) proteins block rapid T4 signaling mediated by αVβ3 [7] suggests that the hormone interacts with the RGD recognition site [44]. Davis *et al*. suggested that, in addition to αVβ3, seven other RGD integrin dimers may function as thyroid hormone receptors [28].

In addition to T4, the iodothyronine T3 binds to integrin αVβ3 and activates both ERK1/2 and phosphatidyl inositol 3-kinase [45]. If T3 interacts with RB αVβ3 to activate ERK1/2 and phosphatidyl inositol 3-kinase, our data indicate that activation of these signaling pathways has no effect on RB I_Na _amplitude. Although we found that T3 does not affect RB I_Na_, previous studies show T3 can increase I_Na _depending on cell type. For example, chronic T3 application increases I_Na _in cultured rat hippocampal neurons [46], but not rat cortex *in vitro*. This result was attributed to increased nuclear thyroid hormone receptor expression in hippocampus versus cortex leading to differential genomic regulation of sodium channel expression. Additionally, T3 rapidly increases I_Na _in cultured myocytes [47,48] through mechanisms that involve rises in intracellular calcium [48] and protein kinase C [47]. Altogether, both T4 and T3 nongenomic signaling result in a variety of downstream consequences due to the diversity of signaling mechanisms activated by plasma membrane thyroid hormone receptors.

### Implications of rapid T4 targeting of Na_v_1.6a and importance to the nervous system

During intrauterine stages, the human embryo requires maternally provided thyroid hormone for normal development [49-51]. However, the specific roles and underlying mechanisms of thyroid hormone action during embryogenesis are poorly understood. Thyroid hormone signals nongenomically to regulate migration of neural cells in the embryonic nervous system [52]. Our data indicate that thyroid hormone, acting rapidly via a plasma membrane receptor, shapes emerging properties of neuronal excitability. Specifically, thyroid hormone rapidly modulates neuronal sodium current by targeting the Na_v_1.6a subunit. The implications for mammals are substantial because the mammalian homologue, Na_v_1.6, shows widespread expression in both the central and peripheral nervous systems [53,54] and is highly expressed during embryonic stages [55]. Moreover, Na_v_1.6a plays important developmental roles for sensory neuron survival and motoneuron axon growth in zebrafish [19,25]. Taken together, these findings indicate that modulation of Na_v_1.6 current during embryonic stages serves as a strategic way to regulate both structural and functional development of the nervous system.

In the context of our results, periods of thyroid hormone deprivation during development would decrease sodium current in neurons expressing both αVβ3 and Na_v_1.6, leading to reduced excitability. Conversely, an excess of thyroid hormone would increase sodium current and potentially induce pathological hyperexcitability, associated with seizures and developmental abnormalities. Interestingly, increased expression of Na_v_1.6 channels are associated with epileptogenesis in mouse hippocampal neurons through mechanisms of enhanced excitability [56], and acute increases in T4 have been reported to cause seizures in humans [57]. Also of note, mice without the functional sodium channel gene *SCN8A *are somewhat resistant to seizures [58] and children with congenital hypothyroidism have a significantly reduced incidence of febrile convulsions [59]. Whether thyroid hormone can acutely influence seizure activity through αVβ3-dependent regulation of Na_v_1.6 in mammals warrants further study. Altogether, T4 regulation of Na_v_1.6a current provides an important mechanism to influence neuronal activity and development.

We focused on rapid T4 signaling in the embryonic nervous system. However, αVβ3 and Na_v_1.6 are also present in the adult nervous system, raising the possibility that T4 acutely regulates sodium current in adults. During adult stages, Na_v_1.6 is the primary sodium channel isoform expressed at nodes of Ranvier [53]. T4 induced modulation of Na_v_1.6 mediated current would alter I_Na _at nodes of Ranvier and, therefore, regulate axonal conductance. The mechanism of thyroid hormone action on adult neurons is unclear, yet alterations in Na_v_1.6 current could result in deficits in sensory neuron axonal conductance and could account for states of hyper- or hypo-reflexia observed in hyper- or hypothyroid patients, respectively [60,61]. Studies on T4-αVβ3's activation of angiogenesis and tumor cell proliferation also have clinical corollaries in adults as hypothyroid states reduce tumor cell proliferation in gliomas [62].

## Conclusion

Our results delineate a pathway for rapid T4 signaling that is initiated by αVβ3 as a T4 receptor, transduced intracellularly by regulation of phosphorylation states, and targets the Na_v_1.6a sodium channel α-subunit. Our proposed pathway predicts that T4's rapid modulation of sodium current requires expression of both αVβ3 and Na_v_1.6a on the responding cell. Our data agree with the prediction in three ways. First, in RBs, T4 rapidly increased sodium current amplitudes at 48 but not 24 hpf, consistent with detection of αVβ3 at 48 but not 24 hpf. Second, upon knock-down of Na_v_1.6a protein, exogenously applied T4 no longer led to a rapid increase in RB I_Na _amplitude. Third, another neuronal population, CaP, which expresses both αVβ3 and Na_v_1.6a, responded to T4 with a rapid increase in I_Na _amplitude. Uncovering the signaling pathways and relevant proteins involved in nongenomic T4 signaling contributes to our understanding of how thyroid hormone regulates development and function of the nervous system.

## Abbreviations

CaP: caudal primary motoneuron; DMSO: dimethyl sulfoxide; ERK1/2: MAPK extracellular regulated kinase; GFP: green fluorescent protein; hpf: hours post-fertilization; I_Na_: sodium current; MAPK: mitogen-activated protein kinase; MO: morpholino; Na_v_: voltage-gated sodium channel; OA: okadaic acid; p38: MAPK p38 isoform; PP: protein phosphatase; RB: Rohon-Beard cell; T3: 3,3',5-triiodo-L-thyronine/triiodothyronine; T4: 3,5,3',5'-tetraiodothyronine or thyroxine; tetrac: 3,3',5,5'-tetraiodothyroacetic acid.

## Competing interests

The authors declare that they have no competing interests.

## Authors' contributions

MAY contributed to experimental design, carried out experiments, and drafted and edited the manuscript. ABR contributed to experimental design and edited the manuscript. All authors read and approved the final manuscript.
